# A decade of HIV care in rural Tanzania: Trends in clinical outcomes and impact of clinic optimisation in an open, prospective cohort

**DOI:** 10.1371/journal.pone.0180983

**Published:** 2017-07-18

**Authors:** Fiona Vanobberghen, Emilio Letang, Anna Gamell, Dorcas K. Mnzava, Diana Faini, Lameck B. Luwanda, Herry Mapesi, Kim Mwamelo, George Sikalengo, Marcel Tanner, Christoph Hatz, Hansjakob Furrer, Manuel Battegay, Tracy R. Glass, on behalf of the KIULARCO Study Group

**Affiliations:** 1 Department of Epidemiology & Public Health, Swiss Tropical & Public Health Institute, Basel, Switzerland; 2 University of Basel, Basel, Switzerland; 3 Ifakara Health Institute, Ifakara, Tanzania; 4 ISGlobal, Barcelona Centre for International Health Research (CRESIB), Hospital Clinic-Universitat de Barcelona, Barcelona, Spain; 5 Bern University Hospital, Bern, Switzerland; 6 University of Bern, Bern, Switzerland; 7 Department of Infectious Diseases and Hospital Epidemiology, Department of Clinical Research, University Hospital Basel, Basel, Switzerland; Tulane University School of Public Health and Tropical Medicine, UNITED STATES

## Abstract

**Objectives:**

Our objectives were to describe trends in enrolment and clinical outcomes in the open, prospective Kilombero and Ulanga Antiretroviral Cohort (KIULARCO) in the Morogoro region of southern Tanzania, and identify strengths and areas for improvement in the care of HIV-positive individuals in rural Tanzania.

**Methods:**

We included adults (≥15 years) and children (<15 years) enrolled in the cohort in 2005–2014. The cohort underwent significant changes from autumn 2012 to optimise care. We evaluated mortality and loss to follow-up (LTFU) using competing risks methods, ART usage, opportunistic infections (OI), co-infections and laboratory abnormalities.

**Results:**

Overall, 7010 adults and 680 children were enrolled; enrolment peaked in 2008 but has increased steadily since 2011. Among adults (65% female; median age 37 [interquartile range 31–45] years), the proportion referred from hospital wards quadrupled in 2013–14 versus earlier years. 653 (9%) adults died and 2648 (38%) were LTFU; the five-year cumulative probabilities of death and LTFU were 10.3% and 44.0%, respectively. Among children, 69 (10%) died and 225 (33%) were LTFU. The corresponding five-year probabilities were 12.1% and 39.6%. Adult ART use (regardless of eligibility) increased from 5% in 2005 to 89% in 2014 (similarly among children), with 9% on second-line therapy in 2014 (17% of children). OI diagnoses increased over time; tuberculosis prevalence at enrolment quadrupled from 6% in 2011 to 26% in 2014. The proportion of newly-enrolled participants assessed for laboratory abnormalities peaked at nearly 100% in 2014 (from a minimum of 24%), yet abnormality prevalences remained fairly constant.

**Conclusions:**

In this cohort, ART usage improved dramatically and is approaching targets of 90%. Improved screening led to increases in detection of OIs and laboratory abnormalities, suggesting that a large number of these co-morbidities previously went undetected and untreated. Further work will address the high LTFU rates and implications for mortality estimates, and the management and outcomes of co-morbidities.

## Introduction

The burden of the HIV epidemic in Tanzania is large, with an estimated 1.5 million adults and children living with HIV in 2012 [1]. The national HIV/AIDS programme was launched in 2004 in response to the rising epidemic [2]. In 2004, the Chronic Diseases Clinic of Ifakara (CDCI) was established within the St Francis Referral Hospital (SFRH), which serves over 600,000 people in the Kilombero and Ulanga districts of the Morogoro region in southern Tanzania [3]. The CDCI was the first rural HIV care and treatment clinic accredited to provide HIV services through the National AIDS Control programme (NACP). Within the framework of the CDCI, a prospective, open cohort was initiated in 2005 by the Ifakara Health Institute, in collaboration with the Swiss Tropical & Public Health Institute and the University Hospital Basel. The Kilombero and Ulanga Antiretroviral Cohort (KIULARCO), with systematic data collection on clinical parameters including antiretroviral therapy (ART) use, co-morbidities and clinical outcomes such as opportunistic infections (OIs) and mortality, plus a plasma biobank spanning ten years, has become a unique research platform.

Since autumn 2012, significant changes have been made at the CDCI targeting education of the clinical staff, optimisation of the patient circuit, implementation of routine diagnostic tests, better integration of HIV services with other units and SFRH wards, and an extensive overhaul of data collection along with the launch of comprehensive electronic medical records. Early evidence suggests that implementation of this broad bundle of services has improved the running of the clinic and enhanced care, such as through earlier and better detection of tuberculosis co-infection [4]. Despite recommendations of increasing CD4 thresholds for ART initiation, most people living with HIV in Tanzania still present late in the course of HIV disease. Late presentation is associated with higher prevalence of OIs and non-AIDS co-morbidities, incidence of immune reconstitution inflammatory syndrome (IRIS) after ART initiation, rate of hospitalisations, use of healthcare resources, and mortality [5–7]. Thus, pre-ART screening of OIs and co-morbidities is a key component to improving outcomes in sub-Saharan Africa. Since 2012, a systematic screening of tuberculosis, cryptococcosis, hepatitis B, syphilis, and renal and liver disease is performed at the CDCI prior to ART initiation.

The aim of this paper is to present the trends over the ten-year data collection period in enrolment and retention in HIV care, mortality, ART usage, and rates of detection of OIs, co-infections and laboratory abnormalities. Our results give a comprehensive overview of the cohort, contribute to the currently-limited knowledge of comorbidities among HIV-positive persons in Tanzania, and identify strengths and areas for improvement in the care of HIV-positive individuals in rural Tanzania.

## Methods

### Study design and population

The CDCI, through the NACP, offers free HIV care and treatment services. Attendees are those identified as HIV-positive following presentation for voluntary counselling and testing (VCT) or through provider-initiated counselling and testing (PITC) while admitted to the SFRH wards, or those who have transferred in from another clinic; all are invited to participate in KIULARCO. For this analysis of prospectively-collected cohort data, we included all patients enrolled from 2005 to 2014, except patients who refused consent; HIV-negative patients; transit patients (enrolled in other clinics but who attended CDCI temporarily, usually for a drug refill while travelling); and patients with missing enrolment or birth dates. We included longitudinal data through to 31 December 2014.

### Clinic procedures and data capture

Since autumn 2012, a number of changes to HIV services were implemented at the CDCI to optimise care. Changes included introduction of PITC in the SFRH wards, and screening of OIS and non-AIDS co-morbidities, including improvements in tuberculosis detection and care with integration of the tuberculosis clinic within the CDCI [4,8]. Routine enrolment screening services now include hepatitis B, syphilis and, for patients with CD4 count <150 cells/mm^3^, cryptococcal antigenaemia using cryptococcal antigen (CRAG) lateral flow assay (IMMY, Norman, Oklahoma) [9]. A One Stop Clinic was created to provide dedicated care for HIV-positive pregnant women, children and their families [10] and HIV pro-viral DNA PCR was implemented for early infant diagnosis.

Since May 2013, the CDCI is paperless; new comprehensive data capture forms were translated into an electronic medical record system where patient data are entered directly during their visit. At registration, we capture sociodemographic data and medical history. During clinical visits, we record vital signs and laboratory results, perform a structured physical examination to determine working diagnoses (captured through ICD-10 codes [11]), and assess treatment adherence, failure and toxicity, and IRIS. ART, cotrimoxazole prophylaxis, isoniazid preventive treatment and fluconazole prophylaxis for CRAG-positive patients are prescribed electronically and dispensed from the clinic pharmacy to the patient or treatment supporter. Visits are scheduled at least every 3 months for those on ART and every 6 months for those not yet on ART.

### Diagnosis of OIs, IRIS, co-infections and laboratory abnormalities among adults

For this study, tuberculosis diagnosis was defined as positive microscopy with acid-fast bacilli, positive Xpert MTB/RIF assay (Cepheid, Sunnyvale, CA, USA) in sputum or other extra pulmonary sample, chest radiograph suggestive of tuberculosis plus at least one symptom, taking anti-tuberculosis medication, or physician diagnosis. Cryptococcosis, Kaposi’s sarcoma, *Pneumocystis jirovecii* pneumonia (PCP), toxoplasmosis, non-Hodgkin’s lymphoma and bacterial pneumonia were physician-diagnosed, with cryptococcosis confirmed by CRAG-positive in plasma and cryptocococcal meningitis in cerebrospinal fluid. IRIS, as diagnosed by the clinician, was categorised as suspected, probable or definite adapting commonly-accepted case definitions [12–15]. Syphilis was diagnosed by Venereal Disease Research Laboratory test and hepatitis B by one surface antigen test. Severe anaemia was defined as haemoglobin <8 g/dL [16], liver impairment as alanine transaminase >2*56 U/L (where 56 U/L is the upper limit of normal) [17], and renal impairment as creatinine clearance <90 ml/min/1.73m^2^, calculated using the Chronic Kidney Disease Epidemiology Collaboration equation [18].

### Statistical methods

Analyses are presented for adults (age ≥15 years at enrolment) and children (<15 years), by enrolment year. After assessing data distributions, similar time periods were grouped together, which also corresponded with changes in cohort leadership. We present participant characteristics at enrolment using appropriate summary statistics. We evaluated the number and proportion of individuals on ART according to first (non-nucleoside reverse transcriptase inhibitor-based) or second line (boosted protease inhibitor-based) therapy, and we describe ART initiations among adults. We assessed mortality and lost to follow-up (LTFU), defined as >60 days passed since their next planned visit [19]. Participants who had not died, were not LTFU and had not transferred out were considered to be in follow-up to 31 December 2014. We estimated time to death or LTFU using competing risks methods [20], with results presented as cumulative incidences and 95% confidence intervals (CI).

Among adults, we evaluated tuberculosis prevalence at enrolment (diagnosed within the first month of enrolment, which may have been previously diagnosed outside of the clinic) and incidence amongst those not positive at enrolment. We evaluated other OI diagnoses following a CD4 count <200 cells/mm^3^. We assessed the testing and diagnosis of laboratory abnormalities within one month of enrolment.

Analyses were performed using Stata version 13 (StataCorp. 2013. *Stata Statistical Software*: *Release 13*. College Station, TX: StataCorp LP).

### Ethical statement

The Ifakara Health Institute Institutional Review Board and the National Health Research Ethics Review Committee of the National Institute for Medical Research of Tanzania provide ethical approval for KIULARCO, including for sample collection, cryopreservation and retrospective analysis. Written informed consent is sought from all participants at registration at the CDCI; for children and adolescents aged <18 years, informed consent is sought from their caregivers. Those who refused consent were excluded from our analyses. Individualised clinical consultations take place in separate rooms at the CDCI, to ensure privacy. Patient-friendly schedules are followed, and each clinician has an assigned pool of patients to improve patient satisfaction and compliance. All data analysed for this study, including laboratory results from samples, were captured under routine care at the CDCI; data are stored on a secure server and were de-identified before analysis.

## Results

### Cohort enrolment

**Fig 1:**
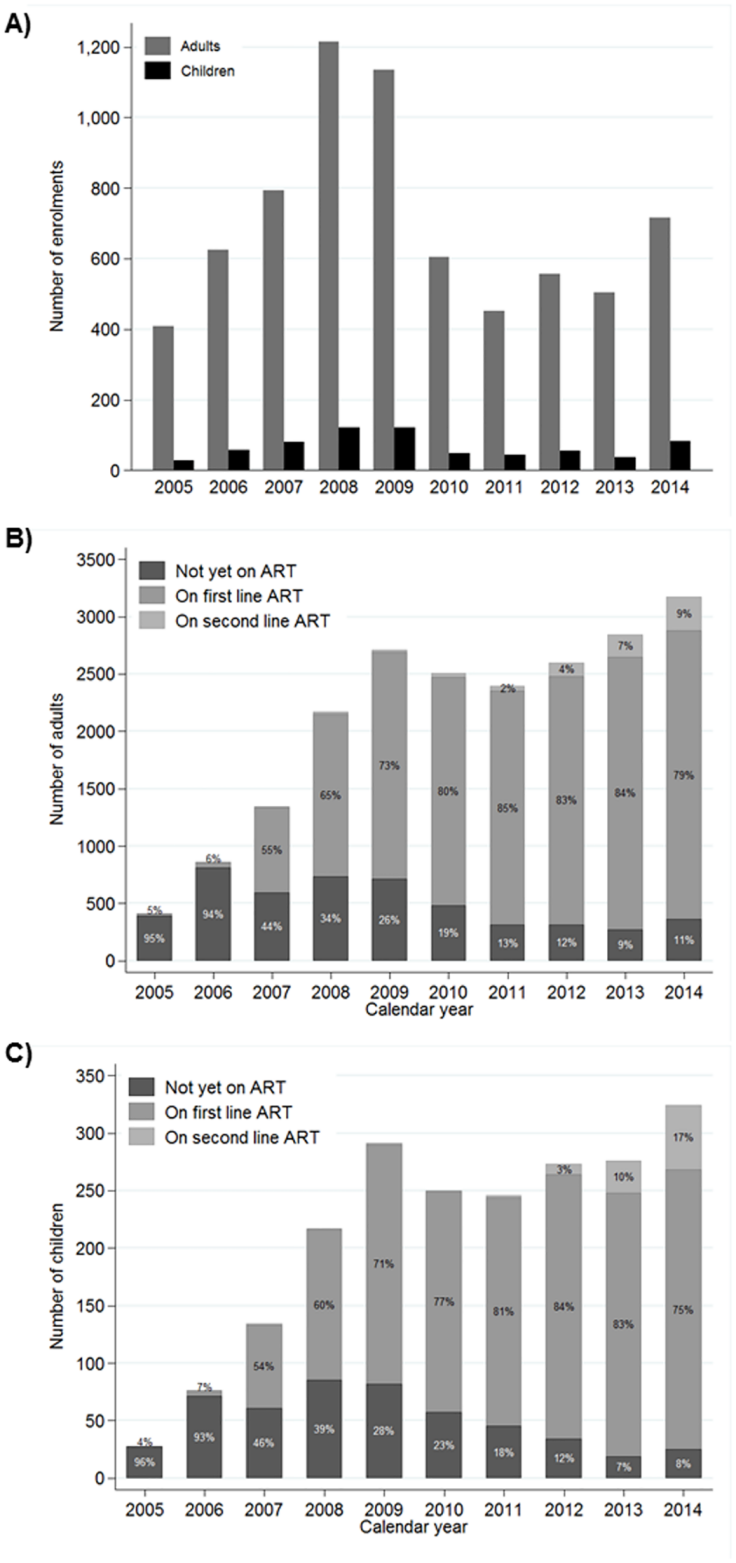
Enrolment and ART usage over time. A) Enrolment. B) ART usage among adults. C) ART usage among children. Adults ≥15 years; children <15 years. Participants on ART at any point in a given year were classified as on ART. Denominator is patients with any clinical or drug visit in a given year. Percentages may not add up to 100% due to rounding. Percentages <1% are not shown.

Of 10,191 individuals, we excluded 1569 (15%) who refused participation, 15 (<1%) subsequently identified as HIV-negative, 779 (8%) transit patients, 121 (1%) who were missing enrolment date and 17 (<1%) who were missing date of birth. Therefore, we included 7010 adults and 680 children in the analysis (S1 Fig). At least 400 participants were enrolled every year since 2005, with a peak in 2008 and a further steady increase over recent years (Fig 1A).

### Participant characteristics at enrolment

The adult sociodemographic characteristics remained fairly stable over time (Table 1), with 42% residing in Ifakara, where the CDCI is situated. Patients mostly came to the CDCI via VCT or self-referral, but in-patient referrals (diagnosed through PITC) and transfers-in rose significantly in 2013–14 while out-patient referrals dropped after 2005–07. The majority of participants were female (65%), were married/cohabiting (53%) and only had at most primary school education (94%). The median (interquartile range [IQR]) age was 37 (31–45) years. Reported disclosure of HIV status to partners and relatives increased respectively from 3% and 2% in 2005–07 to 39% and 65% in 2013–14. Over the same time period, the median time from reported HIV diagnosis to enrolment decreased from 1.6 months (IQR 0.5–7.4) to 0.1 months (0.0–0.4). Trends toward lower WHO stage at enrolment reversed in 2013–14, approaching 2005–07 levels (41% WHO stage III/IV overall), while the proportion of patients with CD4 count <350 cells/mm^3^ remained fairly stable after 2005–07 (69% overall).

**Table 1:** Demographic and clinical characteristics of adults (≥15 years) at enrolment (n = 7010).

	Year of enrolment				
	2005–07	2008–09	2010–12	2013–14	Total
**Number enrolled**	1827 (100%)	2351 (100%)	1611 (100%)	1221 (100%)	7010 (100%)
**Ward**					
Ifakara	632 (35.7%)	880 (37.8%)	901 (56.3%)	498 (40.9%)	2911 (42.1%)
Mngeta	139 (7.9%)	272 (11.7%)	74 (4.6%)	208 (17.1%)	693 (10.0%)
Mang'ula	118 (6.7%)	231 (9.9%)	58 (3.6%)	69 (5.7%)	476 (6.9%)
Mlimba	80 (4.5%)	213 (9.1%)	9 (0.6%)	46 (3.8%)	348 (5.0%)
Other^a^	800 (45.2%)	735 (31.5%)	557 (34.8%)	397 (32.6%)	2489 (36.0%)
**Distance from home to clinic, km**^b^	2 (1–70)	25 (1–81)	1 (1–20)	5 (1–51)	1 (1–51)
**Patient referred from**^c^					
Voluntary counselling and testing or self-referral	956 (52.3%)	1925 (81.9%)	1223 (75.9%)	863 (70.7%)	4967 (70.9%)
Reproductive and child health clinic	69 (3.8%)	113 (4.8%)	136 (8.4%)	53 (4.3%)	371 (5.3%)
Out-patient department	254 (14.6%)	45 (2.0%)	47 (3.1%)	15 (1.2%)	361 (5.4%)
In-patient care hospitalisation (PITC)	24 (1.6%)	75 (3.4%)	47 (3.1%)	125 (10.2%)	271 (4.2%)
Transfer in	0 (0.0%)	31 (1.3%)	44 (2.7%)	213 (17.4%)	288 (4.1%)
Tuberculosis or DOTS	86 (4.7%)	50 (2.1%)	47 (2.9%)	3 (0.2%)	186 (2.7%)
Other	105 (5.7%)	40 (1.7%)	56 (3.5%)	27 (2.2%)	228 (3.3%)
**Sex, female**	1194 (65.4%)	1509 (64.2%)	1038 (64.5%)	816 (66.9%)	4557 (65.0%)
**Marital status, married/cohabiting**	800 (48.4%)	1197 (51.6%)	805 (50.7%)	758 (62.2%)	3560 (52.5%)
**Education, none/primary school only**	497 (94.7%)	766 (94.9%)	805 (93.4%)	1151 (94.3%)	3219 (94.3%)
**Age, years**	37 (31–44)	37 (31–45)	37 (31–45)	37 (31–45)	37 (31–45)
**Pregnant**^d^	26 (3.7%)	62 (6.6%)	88 (10.0%)	76 (10.3%)	252 (7.7%)
**Partner status, HIV-positive**^e^	118 (69.4%)	313 (66.2%)	239 (62.6%)	239 (71.3%)	909 (66.8%)
**Disclosure of HIV status to**^c^					
Partner	60 (3.4%)	152 (6.6%)	354 (24.0%)	358 (38.5%)	924 (14.2%)
Relative	41 (2.3%)	183 (7.9%)	475 (32.1%)	599 (64.5%)	1298 (20.0%)
**Time from HIV diagnosis to enrolment, months**	1.6 (0.5–7.4)	0.5 (0.1–1.3)	0.3 (0.1–0.5)	0.1 (0.0–0.5)	0.4 (0.1–2.0)
**BMI, kg/m**^**2**^					
Underweight (<18.5)	229 (29.6%)	216 (27.9%)	294 (20.6%)	262 (22.6%)	1001 (24.2%)
Normal weight (18.5-<25)	451 (58.3%)	458 (59.1%)	866 (60.7%)	703 (60.7%)	2478 (59.9%)
Overweight (25-<30)	75 (9.7%)	83 (10.7%)	212 (14.9%)	143 (12.3%)	513 (12.4%)
Obese (≥30)	18 (2.3%)	18 (2.3%)	55 (3.9%)	51 (4.4%)	142 (3.4%)
**WHO stage**					
I	596 (38.1%)	779 (33.5%)	653 (41.7%)	433 (39.9%)	2461 (37.6%)
II	246 (15.7%)	666 (28.7%)	347 (22.1%)	160 (14.8%)	1419 (21.7%)
III	448 (28.6%)	603 (25.9%)	431 (27.5%)	296 (27.3%)	1778 (27.2%)
IV	275 (17.6%)	276 (11.9%)	136 (8.7%)	195 (18.0%)	882 (13.5%)
**CD4 count, cells/mm**^**3**^					
<50	85 (15.7%)	216 (14.1%)	117 (14.0%)	179 (17.4%)	597 (15.1%)
50–99	81 (14.9%)	180 (11.7%)	98 (11.7%)	123 (12.0%)	482 (12.2%)
100–199	119 (21.9%)	319 (20.8%)	155 (18.6%)	183 (17.8%)	776 (19.7%)
200–349	117 (21.5%)	322 (21.0%)	188 (22.5%)	219 (21.3%)	846 (21.5%)
350–499	55 (10.1%)	222 (14.5%)	129 (15.4%)	141 (13.7%)	547 (13.9%)
≥500	86 (15.8%)	277 (18.0%)	148 (17.7%)	183 (17.8%)	694 (17.6%)

Results are median (interquartile range) for continuous variables and number (column % of non-missing data) for categorical variables. Variables with >10% missing data: distance from home to clinic (17% missing, since not all wards mapped), education (51% missing, mainly from earlier years), BMI (41% missing, mainly due to missing height), CD4 count (44% missing, mainly from earlier years). CD4 count and WHO stage at enrolment were defined as the measurement closest to the date of enrolment, within -24 to +4 weeks (preference for earlier). BMI, body mass index; DOTS, directly observed treatment, short course; PITC, provider-initiated testing and counselling.

^a^Estimated based on ward of residence.

^b^Wards with <10% of participants each have been combined.

^c^Participants may respond yes to more than one category.

^d^Percentages are of women with non-missing data (missing for 28% women).

^e^Percentages are of those with results (excluding 81% not applicable/missing).

Among children, the median age at enrolment decreased, from 6 (IQR 3–9) years in 2005–07 to 4 years (1–8) in 2013–14, as did the time from HIV diagnosis to enrolment (from 1.0 (0.2–5.4) to 0.1 (0.0–0.5) months; Table 2). Over the same time periods, the percentage of children with WHO stage III/IV increased (from 47% to 77%).

**Table 2:** Demographic and clinical characteristics of children (<15 years) at enrolment (n = 680).

	Year of enrolment				
	2005–07	2008–09	2010–12	2013–14	Total
**Number enrolled**	166 (100%)	244 (100%)	150 (100%)	120 (100%)	680 (100%)
**Sex, female**	72 (43.4%)	115 (47.1%)	74 (49.3%)	56 (46.7%)	317 (46.6%)
**Age, years**	6 (3–9)	4 (2–8)	5 (2–9)	4 (1–8)	5 (2–8)
**Age <5 years**	69 (41.6%)	141 (57.8%)	79 (52.7%)	68 (56.7%)	357 (52.5%)
**Time from HIV diagnosis to enrolment, months**	1.0 (0.2–5.4)	0.3 (0.0–1.3)	0.2 (0.1–1.2)	0.1 (0.0–0.5)	0.3 (0.0–1.6)
**WHO stage**					
I	49 (34.0%)	84 (35.9%)	47 (32.2%)	20 (17.2%)	200 (31.3%)
II	28 (19.4%)	71 (30.3%)	28 (19.2%)	7 (6.0%)	134 (20.9%)
III	39 (27.1%)	66 (28.2%)	54 (37.0%)	50 (43.1%)	209 (32.7%)
IV	28 (19.4%)	13 (5.6%)	17 (11.6%)	39 (33.6%)	97 (15.2%)
**CD4 <25%**	55 (87.3%)	113 (72.4%)	49 (62.8%)	82 (75.9%)	299 (73.8%)

Results are median (interquartile range) for continuous variables and number (column % of non-missing data) for categorical variables. Variables with >10% missing data: CD4% (40% missing, mainly from earlier years). CD4 count and WHO stage at enrolment were defined as the measurement closest to the date of enrolment, within -24 to +4 weeks (preference for earlier).

### ART usage

Overall, 4989 (71%) adults and 513 (75%) children ever received ART under the care of the CDCI, of whom 484 (10%) and 41 (8%), respectively, had prior ART exposure. Of adults attending the CDCI in any given year, the percentage of patients on ART (regardless of eligibility) rapidly increased from 5% in 2005 to at least 80% since 2010 (Fig 1B), with similar patterns among children (Fig 1C). The proportion of adults on second line therapy increased from none pre-2008 to 9% in 2014, while the proportion of children on second line therapy increased rapidly from ≤1% pre-2012 to 17% in 2014. Among 361 adults not on ART in 2014, 191 (53%) were not yet eligible for therapy. Of the remaining 170 adults, 132 (37%) had been under follow-up for <90 days, 1 (<1%) did not have eligibility information captured, and 37 (22%) had been under follow-up for at least 90 days and were eligible for ART but had not yet started.

### ART initiations among adults

Overall, 4502 adults initiated ART (excluding women starting for prevention of mother-to-child HIV transmission) for the first time at the CDCI (Table 3). In 2005–2007, 70% of patients initiated on stavudine, lamivudine and nevirapine. Over time, efavirenz replaced nevirapine, the backbone of tenofovir disoproxil fumarate with emtricitabine or lamivudine became more common (80% of initiations in 2013–14), and stavudine was phased out, in line with national guidelines [21]. There were some albeit small increases over time in the proportion of participants initiating ART with CD4 count ≥350 cells/mm^3^, at 16%, 11%, 17% and 19% in 2005–07, 2008–09, 2010–12 and 2013–14, respectively.

**Table 3:** Characteristics of adults (≥15 years) at ART initiation.

	Year of ART initiation				
	2005–07	2008–09	2010–12	2013–14	Total
**Number initiated ART**	679 (100%)	1547 (100%)	1358 (100%)	918 (100%)	4502 (100%)
**Time from enrolment to ART initiation, months**^a^	4.7 (0.9–12.6)	1.0 (0.2–4.1)	0.5 (0.1–8.1)	0.5 (0.2–2.5)	0.9 (0.2–6.2)
**ART regimen started**					
d4T+3TC+NVP	474 (69.8%)	915 (59.1%)	160 (11.8%)	0 (0.0%)	1549 (34.4%)
d4T+3TC+EFV	22 (3.2%)	45 (2.9%)	9 (0.7%)	0 (0.0%)	76 (1.7%)
AZT+3TC+NVP	7 (1.0%)	22 (1.4%)	125 (9.2%)	53 (5.8%)	207 (4.6%)
AZT+3TC+EFV	172 (25.3%)	503 (32.5%)	490 (36.1%)	118 (12.9%)	1283 (28.5%)
TDF+FTC+EFV	0 (0.0%)	0 (0.0%)	239 (17.6%)	352 (38.3%)	591 (13.1%)
TDF+3TC+EFV	0 (0.0%)	0 (0.0%)	0 (0.0%)	382 (41.6%)	382 (8.5%)
Other first line	0 (0.0%)	0 (0.0%)	3 (0.2%)	3 (0.3%)	6 (0.1%)
Second line (boosted PI-based)	2 (0.3%)	0 (0.0%)	7 (0.5%)	10 (1.1%)	19 (0.4%)
Other^b^	2 (0.3%)	62 (4.0%)	325 (23.9%)	0 (0.0%)	389 (8.6%)
**CD4 count at initiation, cells/mm**^**3**^					
<100	134 (26.7%)	412 (35.8%)	208 (26.4%)	249 (31.1%)	1003 (31.0%)
100–199	162 (32.3%)	355 (30.9%)	187 (23.7%)	166 (20.8%)	870 (26.9%)
200–349	125 (25.0%)	262 (22.8%)	262 (33.2%)	236 (29.5%)	885 (27.3%)
350–499	45 (9.0%)	66 (5.7%)	74 (9.4%)	91 (11.4%)	276 (8.5%)
≥500	35 (7.0%)	55 (4.8%)	58 (7.4%)	58 (7.2%)	206 (6.4%)

Results are median (interquartile range) for continuous variables and number (column % of non-missing data) for categorical variables. Variables with >10% missing data: CD4 count (26%, 26%, 42% and 16% missing in 2005–07, 2008–09, 2010–12, 2013–14, respectively). CD4 count at ART initiation was defined as the measurement closest to the date of initiation, within-24 to +4 weeks (preference for earlier). d4T, stavudine; 3TC, lamivudine; NVP, nevirapine; EFV, efavirenz; AZT, zidovudine; TDF, tenofovir disoproxil fumarate; FTC, emtricitabine.

^a^This table is restricted to participants who were observed to initiate ART, and therefore does not take into account participants who were not (yet) observed to initiate ART.

^b^Regimen unknown therefore not possible to classify as first or second line.

### Mortality and loss to follow-up

**Fig 2:**
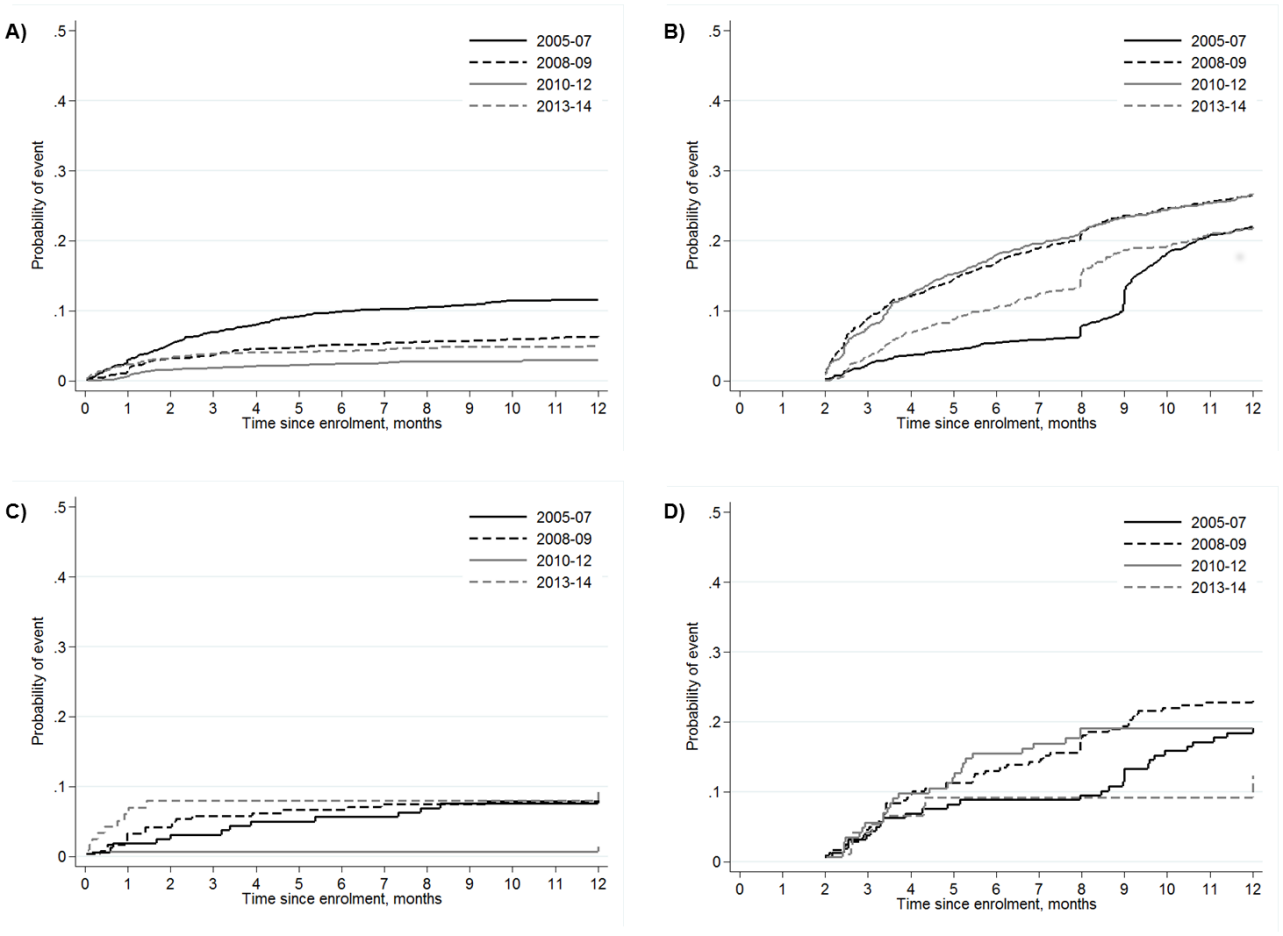
Incidence of death and loss to follow up (LTFU). A) Adults: Death by enrolment year. B) Adults: LTFU by enrolment year. C) Children: Death by enrolment year. D) Children: LTFU by enrolment year. Adults ≥15 years; children <15 years. Analysis by competing risks. Participants were considered LTFU if more than 60 days had passed since their next planned visit (based on next appointment date, or otherwise assumed to be every 3 months for those on ART and every 6 months for those not yet on ART, not including those known to have died or transferred out). Only the first 12 months since enrolment are shown (since little further follow-up was available for those enrolled in 2013–14), and the y-axes are truncated at 0.5.

Over approximately 18,000 person-years at risk, 653 (9%) adults died and 2648 (38%) were LTFU (S1 Fig). By five years after enrolment, the cumulative probability of death was 10.3% (95% CI 9.5–11.1) and LTFU was 44.0% (42.7–45.4). By year of registration, the probabilities of reported death at 12 months after enrolment were 11.6% (10.2–13.1), 6.3% (5.4–7.4), 3.0% (2.2–3.9) and 5.2% (3.9–6.8) among those registered in 2005–07, 2008–09, 2010–12 and 2013–14, respectively (Fig 2A). The corresponding values for LTFU were 22.0% (20.1–24.0), 26.5% (24.7–28.4), 26.7% (24.5–28.9) and 21.9% (19.0–24.9; Fig 2B).

Over approximately 1900 person-years at risk, 69 (10%) children died and 225 (33%) were LTFU (S1 Fig). By five years after enrolment, the cumulative probability of death was 12.1% (9.5–15.1) and LTFU was 39.6% (35.2–43.9). Over the same time periods as above, the probabilities of reported death by 12 months after enrolment were 8.2% (4.6–13.1), 8.4% (5.3–12.3), 1.4% (0.3–4.5) and 9.4% (4.7–15.9), respectively (Fig 2C). The corresponding values for LTFU were 19.0% (13.3–25.5), 23.3% (18.1–28.8), 19.1% (13.1–26.0) and 12.3% (5.5–22.1; Fig 2D).

Among both adults and children, the earliest that one can meet the LTFU definition is two months after the next planned visit (Fig 2B and 2D). The larger increases in LTFU at 8 months among adults enrolled in some of the enrolment years are attributable to those who did not start ART at enrolment and did not return to the clinic for a follow-up visit. Among 6515 adults who were ART-naïve before enrolment, the percentage starting ART within 2 weeks of enrolment increased over time (from 7% in 2005–07 to 41% in 2013–14), and those starting ART within this period were less likely to be LTFU within the first year after enrolment than those not starting ART (20% versus 25%; Χ^2^ test, p<0.001). Across all participants, 902 (12%) had a gap in care of >1 year before re-engaging in care (ie returning for a clinic visit), and 487 (6%) had a gap of >2 years.

### Opportunistic infections and IRIS among adults

**Fig 3:**
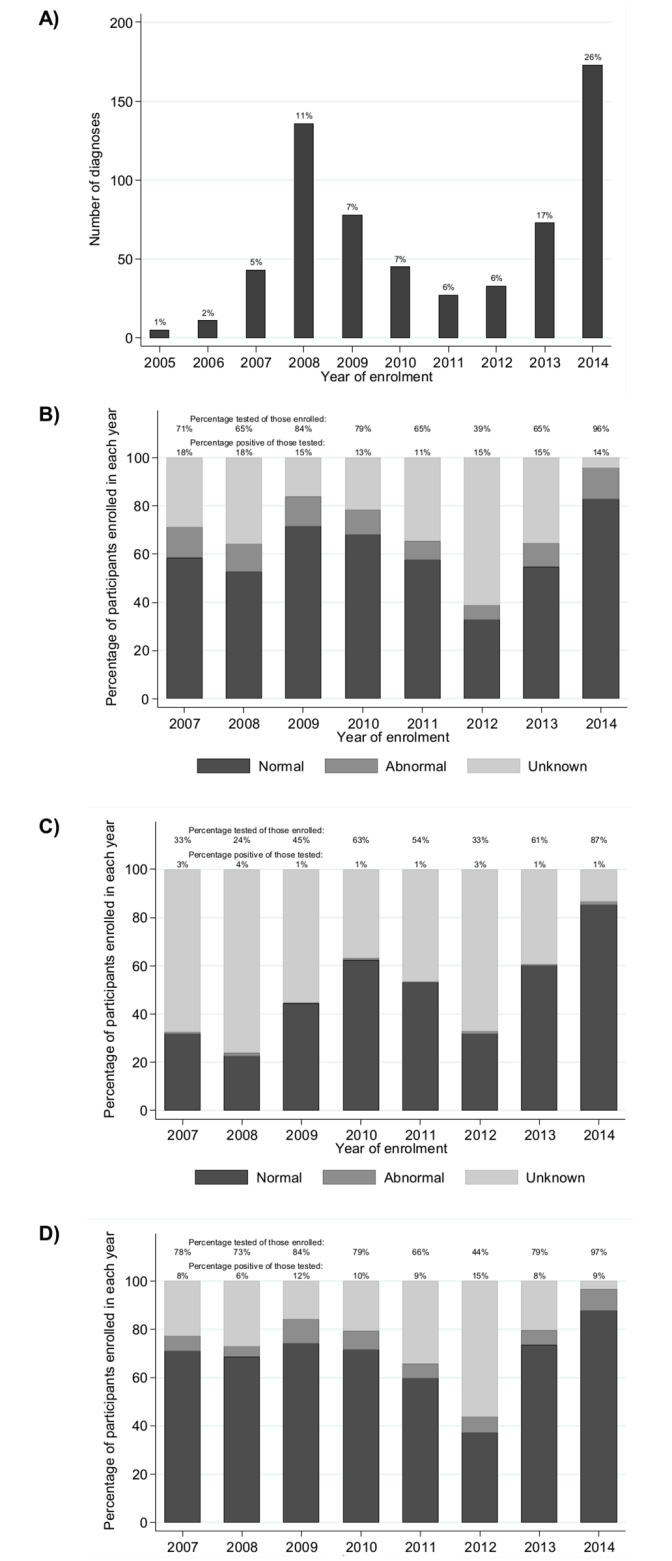
Tuberculosis and laboratory abnormalities at enrolment among adults (≥15 years). A) Diagnosis of tuberculosis. B) Testing and diagnosis of severe anaemia. C) Testing and diagnosis of liver impairment. D) Testing and diagnosis of renal impairment. For tuberculosis, values show the percentage of diagnoses within the CDCI (within the first month following enrolment; these OIs may have been previously diagnosed outside of the clinic; with denominator the number of participants who had a clinical visit within the first month following enrolment in that year). For laboratory tests, no tests were performed in 2005 and few in 2006 therefore these years were omitted. A window of-24 to +4 weeks relative to registration date was allowed, with preference for before. Severe anaemia defined as haemoglobin <8 g/dL [16]. Liver impairment defined as alanine transaminase >2*56 U/L, ie twice the upper limit of normal [17]. Renal impairment defined as creatinine clearance <90 ml/min/1.73m^2^, calculated using the Chronic Kidney Disease Epidemiology Collaboration equation [18].

The prevalence of tuberculosis diagnosis among adults at enrolment peaked at 11% in 2008, declined and then rapidly increased after 2012 to 26% in 2014 (Fig 3A). Among those without tuberculosis at enrolment, the incidence peaked at 8% in 2008 and has remained at 2–4% since then.

Before 2011, diagnoses of disseminated cryptococcosis, Kaposi sarcoma and PCP were often not recorded; among adults with results captured at their first CD4 count <200 cells/mm^3^, the annual prevalences were typically <1%. However, in 2014, the prevalences were 5.0%, 3.6% and 5.8%, respectively. Toxoplasmosis, non-Hodgkin’s lymphoma and bacterial pneumonia were not consistently recorded before 2013; the prevalences among adults with first CD4 count <200 cells/mm^3^ in 2014 were 2.2%, 0.5% and 8.9%, respectively.

IRIS detection was low pre-2013 (≤5 diagnoses per year), and increased to 39 (1.7% of adults on ART without previous IRIS) and 49 (1.9%) diagnoses in 2013 and 2014, respectively. In 2013–14, the classification of diagnoses was 58 suspected, 18 probable and 11 definite.

### Co-infections among adults

Few syphilis presumptive diagnoses were made before 2014 (≤12 per year), but 133 new diagnoses were made in 2014 (16% of adults with a visit and not previously positive). Hepatitis B was not routinely captured before 2013, but as the result of a screening initiative in 2014, 199 (7%) new diagnoses of chronic hepatitis B were made in 2014 [22].

### Laboratory abnormalities among adults

The proportion of newly-enrolled participants assessed for laboratory abnormalities peaked in 2014 following implementation of routine screening, approaching 100% coverage (96%, 87% and 97% for anaemia, liver impairment and renal impairment, respectively; Fig 3B–3D). Prevalences of severe anaemia, liver impairment and renal impairment remained between 11–18%, 1–4% and 6–15%, respectively, despite large increases in the proportion tested.

## Discussion

In this comprehensive longitudinal analysis of a prospective cohort spanning 10 years and including nearly 8000 HIV-positive patients, we found that a bundle of measures aiming to improve patient care have led to better linkage to care within the hospital, shortening of time between diagnosis and enrolment, increases in ART usage, improved screening of OIs, and higher testing and detection of laboratory abnormalities.

Until 2011, the CDCI observed enrolment patterns similar to national trends [2], but thereafter–in contrast to national trends–enrolment began to steadily increase. We attribute the recent increases to the 2014 implementation of consistent PITC in the medical, paediatric and tuberculosis wards, and the out-patient department of SFRH; average monthly enrolment increased from 45 in 2013 to 67 in 2014. Similar enrolment patterns were observed in children, despite being more difficult to diagnose and link to care [23]. The average monthly enrolment increased from three children per month in April-December 2013 to eight in the same time period in 2014 after the launch in early 2014 of the One Stop Clinic, and implementation of PITC and HIV pro-viral DNA PCR for infant diagnosis. In recent years, we found that patients were linking to care sooner following their HIV diagnosis, in part attributable to measures such as the national VCT campaign in 2007–08 [24]. However, we did not observe corresponding improvements in disease stage or CD4 count at enrolment, likely related to the implementation of PITC capturing sicker patients, and suggesting that people are still waiting too long to get tested and presenting late in the course of the disease. Our findings are in line with the national data, where approximately 45% of people with a CD4 count result at enrolment had <200 cells/mm^3^ [2].

ART usage increased dramatically over time, despite Tanzanian guidelines not recommending universal ART initiation for those with CD4 counts >350 cells/mm^3^ at the time of this study [25] and little evidence of changes in CD4 count distributions over time. Our observed rates of ART usage of 89% among adults and 92% among children in 2014 are substantially higher than the reported national coverage (61% and 26% of eligible adults and children, respectively, in 2012) [26] and are in line with UNAIDS treatment targets for 2020 [23]. At the CDCI in 2014, eligibility to start ART with respect to CD4 count was evaluated in 94% of patients at enrolment compared to a maximum of 70% in earlier years and 27% nationwide in 2011 [25], likely due to our improved clinic circuit and electronic data capture. This accelerated process for evaluating eligibility should lead to reductions in loss to follow up, which was 25% by one year in those who did not start ART shortly after enrolment, suggesting they were linked to but failed to remain in care. In light of the results of the START and TEMPRANO trials [27,28], moving to a test-and-treat approach will not only lead to earlier engagement in care, but dramatically improve patient outcomes.

Over time, the absolute numbers of diagnoses of OIs and laboratory abnormalities has increased, most notably in 2013–14. This can be attributed to an increasing number of laboratory tests being performed–in part related to growth in equipment and expertise, policy changes regarding screening at baseline, and improved medical evaluations, training and data capture. Tuberculosis diagnoses at enrolment quadrupled from pre-2011 to 2014, most likely attributable to improvements in detection rather than underlying changes in prevalence, and indicating that infections may have remained undiagnosed in earlier years [4]. Systematic laboratory-based CRAG screening among those with low CD4 count, followed by lumbar puncture to detect meningitis, allowed reliable ascertainment of disseminated cryptococcosis and cryptococcal meningitis [29]. Our observed prevalence in 2014 of 5% is consistent with other studies (3–7.1% [30]). While globally the incidence of Kaposi sarcoma is decreasing with increasing ART coverage [31,32], we observed increasing prevalence over time, reaching 3.6% among patients enrolled in 2014 with low CD4 count, most likely due to improved detection, although the limitations of physician-diagnosis are known [33]. Among the same KIULARCO patients, PCP prevalence was lower than in a recent meta-analysis of studies conducted between 1995 and 2015 (5.8% versus 15.4%) but the authors found substantial heterogeneity by time, clinical setting and diagnostic method [34]. Our prevalence was also lower than a study from 2006 (10.4%), however this was in HIV-infected patients presenting with cough [35]. Further, our estimates should be interpreted as upper limits, since diagnostic tools for the etiologic diagnosis were not in place. Importantly, toxoplasmosis, non-Hodgkin’s lymphoma and bacterial pneumonia were not investigated prior to 2012, and the number of diagnoses of bacterial pneumonia in particular indicates that there was previously a large unrecognised and hence untreated problem. Toxoplasmosis was suspected on the basis of focal palsies in patients with low CD4 count, but rarely confirmed through image techniques such as CT scan, which is not available in Ifakara. Some studies report declining incidence of non-Hodgkin’s lymphoma with increasing ART coverage [32], but a study from Uganda found increasing incidence [31]; we observed low prevalence (<1%), which should be cautiously interpreted due to the lack of histologic confirmation. In addition, bacterial pneumonia was not confirmed with pneumococcal antigen or blood cultures but refers to acute respiratory infections with radiologic consolidation that responded to antimicrobial therapy.

Measuring haematological, renal and liver parameters can aid in choosing the most appropriate ART regimen to minimise drug toxicity; tenofovir disoproxil fumarate is associated with renal impairment, zidovudine with haematological abnormalities, and nevirapine and efavirenz with liver toxicity, among others [36–43]. In Tanzania, current guidelines recommend haematological and biochemistry renal and liver function tests pre-ART and at ART initiation, and haemoglobin, creatinine and ALT monitoring for those on zidovudine, tenofovir disoproxil fumarate and nevirapine respectively [44]. The associated financial burden is likely to be a contributory factor for the high rates of early attrition in some African settings [45]. In the CDCI, these services are provided at no cost to the patient and coverage of baseline laboratory testing increased significantly over time to reach approximately 90% in 2014. However, the prevalence of laboratory abnormalities did not change substantially. Based on this, we estimate that there were 37, 3 and 22 undetected cases of severe anaemia, liver and renal impairment at enrolment, respectively, in 2012 alone (representing 7%, 1% and 4% of adults enrolled in that year, respectively).

The observed trends in death and LTFU are more difficult to interpret. There were clear differences in the death rates by year of enrolment. In sub-Saharan Africa, the adult mortality rate in the first year after treatment initiation is estimated to be in the range 8–26% [46], therefore the observed low rate in our cohort in 2010–12 in particular is implausible and suggests that perhaps a large proportion of deaths were not being captured. One-year LTFU rates in adults remained between 22–27% over time but showed trends toward improvement in 2013–14. The higher observed death rate in children during the first months under care in 2013–14 can likely be attributed to the higher proportions presenting with WHO stage III/IV, due to improved clinical assessment and higher proportions of children identified through PITC in the hospital wards [10]. Our high LTFU rates are in line with similar cohorts [47] and may partly be explained by silent transfers to other clinics [48]. Regardless, such LTFU presents challenges in obtaining accurate mortality estimates. Time-dependent modelling taking into account LTFU rates over time is the focus of on-going work [49].

The impact of the clinic changes has led to improvements in patient care across a broad range of outcomes, and the benefits are felt by both staff and patients, with a decrease in lost test results files and improved efficiency in running the clinic. However, the implementation of these changes has not been without challenges. The increased ascertainment of HIV through the consistent implementation of PITC has led to an overburdened clinic with long waiting times and intensified demand for human resources. Further, there is a shortage of skilled and HIV-experienced healthcare workers and we face difficulties retaining them in peripheral rural settings. Moreover, structural system barriers lead to frequent shortages of reagents, antiretrovirals and other drugs for OIs, thus impairing patients’ outcomes. Finally, developing and maintaining an electronic medical record system in this setting has been hampered by limitations in infrastructure, power cuts, and a lack of local technological expertise. Strengths of the cohort are the large number of patients enrolled over the last decade, with the accompanying biobank, and in recent years the data captured are extensive.

In summary, we have illustrated what can be achieved by a clinic delivering HIV care and treatment services to a rural population of around 600,000 people. Since its inception over ten years ago, the CDCI has undergone substantial changes; a number of improvements in patient care and outcomes have already been observed but the full implications of the changes remain to be seen. Future implementation of routine viral load monitoring and optimisation of patient tracking will also improve care and provide a more accurate picture of impact. These developments and enhancements to data capture, allowing us to provide for example detailed data on OIs and laboratory abnormalities for which there are limited reports, together with the biobank, yield a rich resource which can be applied to address public health questions of direct relevance to inform further improvements in patient care.

## Supporting information

S1 Fig Flowchart of participants in the cohort. (TIF)
