# Supplementary figures and images for: A decade of HIV care in rural Tanzania: Trends in clinical outcomes and impact of clinic optimisation in an open, prospective cohort
Source: PLoS One. 2017 Jul 18;12(7):e0180983. doi: 10.1371/journal.pone.0180983 (PMC5515476; doi:10.1371/journal.pone.0180983)

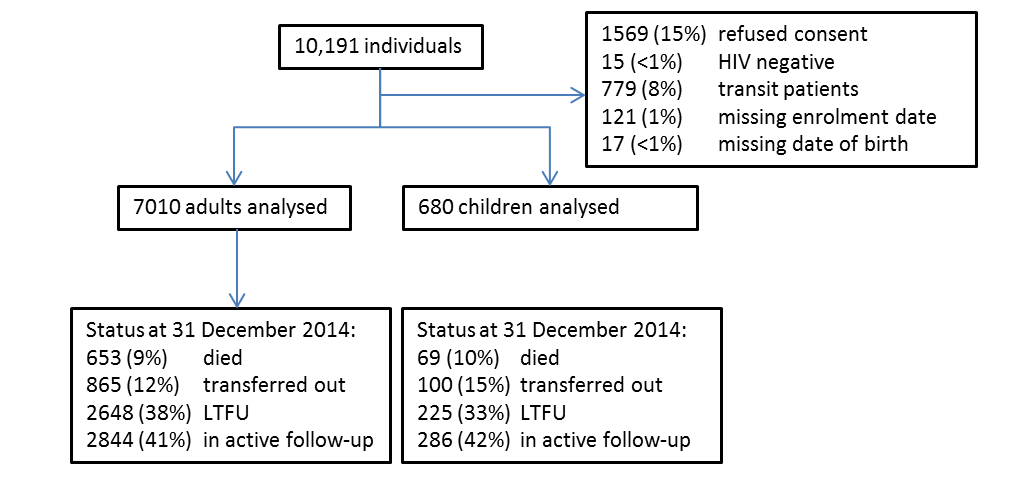

Supplement: S1 Fig — (TIF) [file pone.0180983.s001.tif]
